# Bulk plasmon-polaritons in hyperbolic nanorod metamaterial waveguides

**DOI:** 10.1002/lpor.201400457

**Published:** 2015-04-09

**Authors:** Nikolaos Vasilantonakis, Mazhar E Nasir, Wayne Dickson, Gregory A Wurtz, Anatoly V Zayats

**Affiliations:** Department of Physics, King's College LondonStrand, London, WC2R 2LS, UK

**Keywords:** Plasmonics, waveguides, Metamaterials, Hyperbolic

## Abstract

Hyperbolic metamaterials comprised of an array of plasmonic nanorods provide a unique platform for designing optical sensors and integrating nonlinear and active nanophotonic functionalities. In this work, the waveguiding properties and mode structure of planar anisotropic metamaterial waveguides are characterized experimentally and theoretically. While ordinary modes are the typical guided modes of the highly anisotropic waveguides, extraordinary modes, below the effective plasma frequency, exist in a hyperbolic metamaterial slab in the form of bulk plasmon-polaritons, in analogy to planar-cavity exciton-polaritons in semiconductors. They may have very low or negative group velocity with high effective refractive indices (up to 10) and have an unusual cut-off from the high-frequency side, providing deep-subwavelength (*λ*_0_/6–*λ*_0_/8 waveguide thickness) single-mode guiding. These properties, dictated by the hyperbolic anisotropy of the metamaterial, may be tuned by altering the geometrical parameters of the nanorod composite.

## 1 Introduction

Light interacts with a resonant medium by forming polaritonic waves. These are mixed excitations of the electromagnetic field (photons) with quasiparticles related to material resonances. These quasiparticles include phonons, excitons in semiconductors and plasmons in conductors 1 or atomic ensembles 2. Exciton-polaritons are the most intensely studied having numerous applications in semiconductor lasers. When electromagnetic fields propagate in resonant media, polaritonic waves are formed and their behavior is governed by the dispersion determined by the material resonances around which negative permittivity can be observed 1. Both phonon-polariton and exciton-polaritons can exist in the bulk of the material and at the interface with the adjacent medium as surface electromagnetic waves (surface polaritons). On the other hand, at frequencies lower than the electron plasma frequency, usually only surface plasmon-polaritons (SPPs) have been considered, since electric fields do not significantly penetrate metals over more than the skin depth 3.

Plasmonic nanorod metamaterials possess unique optical properties 2 making them unrivalled for applications in imaging 4, sensing 5,6, ultrasound detection 7, designing nonlinear optical properties 8–10 and controlling quantum optical processes 11,12. These metamaterials exhibit hyperbolic isofrequency surfaces in a spectral range where the real part of the diagonal components of the permittivity tensor, corresponding to ordinary and extraordinary axis, have opposite signs. The hyperbolic dispersion allows various unusual modes to exist with very high wavevectors and negative group velocity, opening up new degrees of freedom for the development of both integrated and free-space optical functionalities.

In this article, the behavior of bulk plasmon-polaritons in a planar slab of an anisotropic metamaterial is studied both experimentally and theoretically. It is found that such extraordinary modes exhibit low or negative group velocity and low group-velocity dispersion (GVD). Furthermore, a peculiar high-frequency cut-off for transverse-magnetic (TM) polarized modes allows subwavelength single-mode guiding with *λ*_0_/6–*λ*_0_/8 waveguide thickness, in contrast to ordinary, transverse-electric (TE) modes which behave as in conventional transparent dielectric waveguides. Bulk plasmon-polaritons of an anisotropic plasmonic metamaterial slab can be considered in analogy to exciton-polaritons in a semiconductor cavity. Such bulk plasmon-polariton modes can be tailored by controlling the geometry of the metamaterial design, and their properties can be utilized in applications requiring sensitive control over both group- and phase-velocity dispersion, such as waveguides, sensors or nonlinear optical devices.

The article is structured as follows. Section describes the theory and numerical simulations of the mode structure of a metamaterial slab: in Section, the effective permittivity of the anisotropic metamaterial is described and the notion of effective plasma frequency is introduced; in Section, the mode structure of the metamaterial slab is studied both numerically and with an approximate analytical formulation. Section describes experimental studies of the metamaterial's modes.

## 2 Theoretical formulation

### 2.1 Optical properties of plasmonic nanorod composites

**Figure 1:**
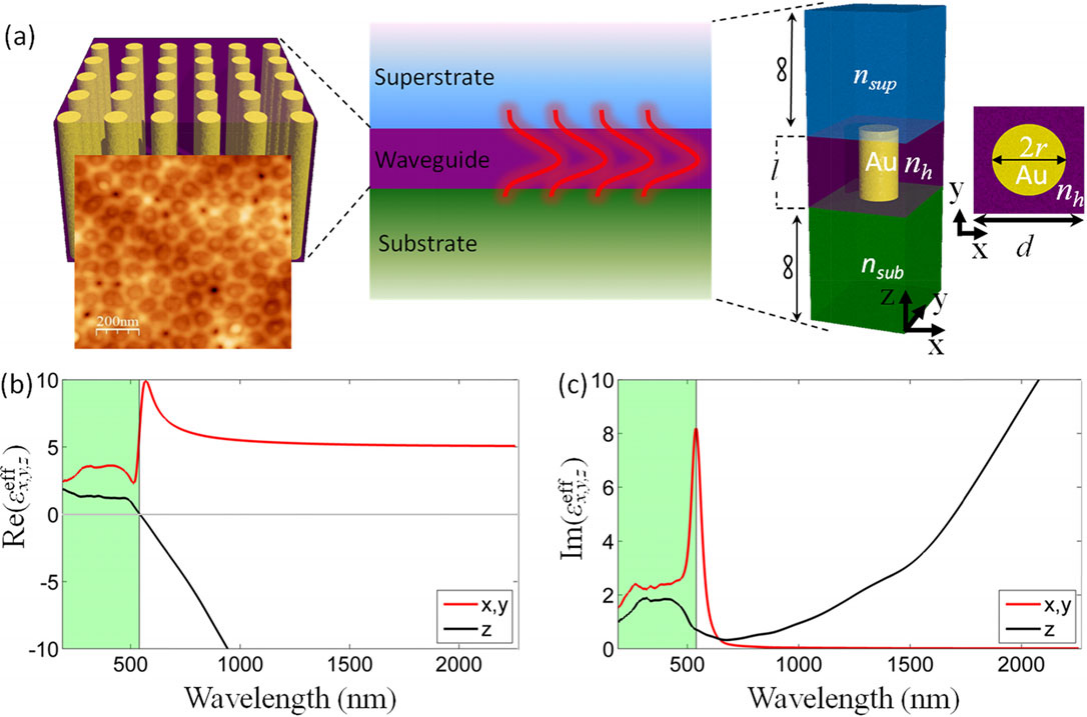
(a) Schematics of the planar metamaterial waveguide geometry consisting of an array of Au nanorods. Left: Schematic of the metamaterial's internal structure and top-view atomic force microscopy image of the metamaterial used in the experiment (topography variations are less than 10 nm). Center: effective medium representation of the metamaterial slab placed between a semi-infinite substrate (*n*_sub_) and a superstrate (*n*_sup_). Right: geometry and cross section of the unit cell of the metamaterial used in the numerical simulations. (b) and (c) Spectral dependences of (b) real and (c) imaginary parts of the effective permittivity of the metamaterial along ordinary and extraordinary directions for *p* = 0.32. Elliptic and hyperbolic dispersion ranges are shown with green and white background colors, respectively.

We consider a planar metamaterial waveguide formed by a finite thickness slab of aligned gold (Au) nanorods (Fig.1a). The anisotropic optical properties of the metamaterial can be described in the effective medium theory (EMT) by a diagonal permittivity tensor with nonzero components 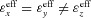, which can be expressed in the Maxwell–Garnet approximation 13 as

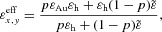
1

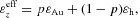
2where 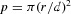 is the nanorod concentration, with *r* being the nanorod radius and *d* being the period of a square lattice, ε_Au_ and ε_h_ are the permittivities of the gold nanorods 14 and the embedding porous alumina (AAO) medium (ε_h_ = 2.56), respectively, and 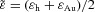. Depending on the geometrical parameters of the metamaterial and the wavelength range, either elliptical with 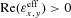 and 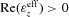 or hyperbolic with 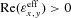 and 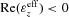 dispersion can be observed (Fig.1b). In fact, by selecting specific geometrical parameters, hyperbolic dispersion can be achieved throughout the visible and near-infrared spectral ranges. The spectral dependence of the imaginary part of the permittivities follows a typical behavior for a resonant dielectric and an electron plasma along the ordinary (*x*,*y*) and the extraordinary (*z*) directions, respectively (Fig.1c).

We have introduced the effective plasma frequency of the metamaterial to characterize their metal-like behavior for TM-polarized fields via the free-electron Drude model 15: 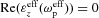. Note that this consideration is not equivalent to the homogenization of the electron density in metallic components over the metamaterial volume, which would not reflect the physical processes by eliminating anisotropy. Using the effective medium parameters (Eqs. 1 and 2, the effective plasma frequency can be derived as 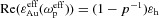. An approximate expression for the effective plasma frequency can then be obtained assuming a lossless Drude-like permittivity for Au given by 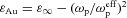, where 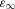 is the high-frequency background and ω_p_ is the free-electron plasma frequency of Au:

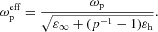
3

Equation 3 shows that the metamaterial's effective plasma frequency can be tuned by varying the permittivity of the host medium and/or concentration of nanorods. Note that for a concentration *p* = 1, corresponding to a homogeneous and isotropic metal layer, the plasma frequency of the bulk metal is recovered.

**Figure 2:**
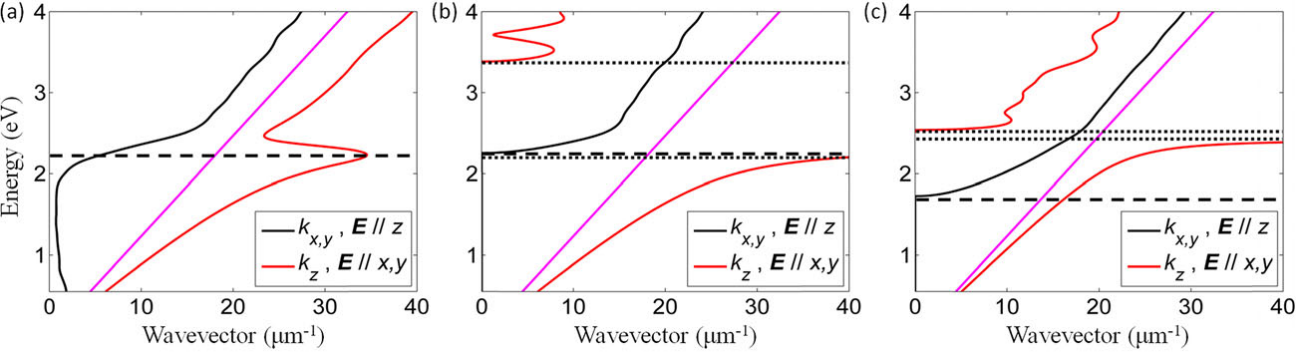
Plasmon-polariton dispersions in an infinite Au nanorod metamaterial in ordinary and extraordinary directions: (a) the metamaterial parameters are as in Fig.1, (b) the same as in (a) but with losses artificially reduced 100 times to show the asymptotic behavior, (c) the same as in (b) but for *p* = 0.13. The light line in AAO (magenta) is also shown. The dashed line shows the effective plasma frequency for extraordinary waves. The EVL regime takes place between the dotted lines.

In the case of a spatially infinite anisotropic material, invariant in the *z*-direction, the electromagnetic wave dispersion can be plotted for both ordinary and extraordinary waves (Fig.2a). As expected, the ordinary wave has a typical dispersion for a transparent dielectric, lying to the right of the light line in the AAO matrix since the effective refractive index is increased due to the presence of metal, with the resonance–the so-called epsilon-very-large (EVL) regime 16,17–determined by cylindrical surface plasmon (CSP) excitations on the rods 18. At the same time, the dispersion of extraordinary waves is that of a typical Drude-like metal with an effective plasma frequency 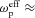 2.2 eV corresponding to the metamaterial's transition from the elliptic to the hyperbolic regime. It is instructive to consider a “gedanken” situation with reduced losses in Au that allows the nature of the dispersion properties of ordinary and extraordinary waves to be clearly revealed (Fig.2b). In the low-loss case, there is a range of frequencies where 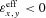 in the previously discussed EVL regime, where a bandgap opens in the TE-mode dispersion. This TE-mode bandgap and the effective plasma frequency for the TM mode can be tuned with the geometry of the metamaterial realization. While for low nanorod concentration, at least one of the mode is always present and the metamaterial behaves either as an elliptic or as an hyperbolic medium, for higher concentrations (*p* > 0.28) and low loss, metallic behavior can be observed in the frequency range where all diagonal components of the effective permittivity tensor are negative simultaneously, 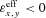 and 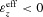, resulting in the disappearance of bulk modes (Fig.2c). This regime, however, takes place in the wavelength range and for loss parameters where nonlocal, spatial dispersion effects occur and that requires a different theoretical treatment 18,19.

**Figure 3:**
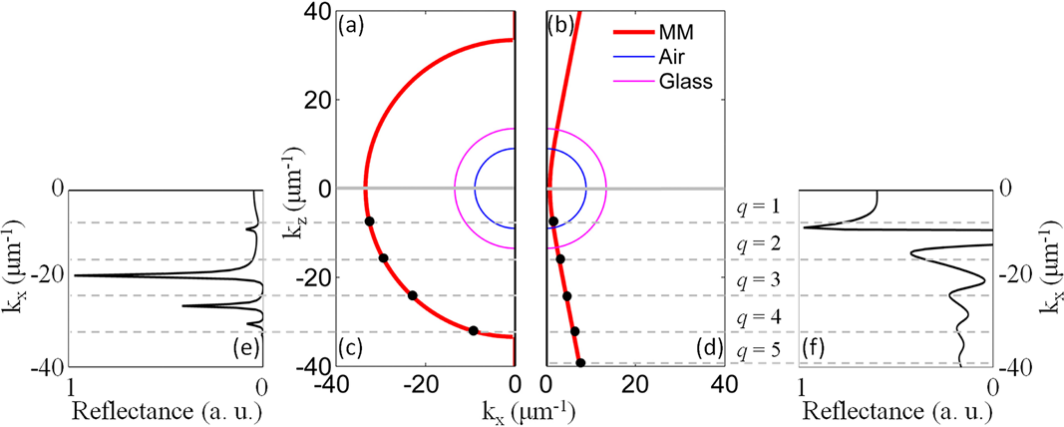
(a) and (b) Isofrequency contours in the first Brillouin zone calculated for a frequency corresponding to a free-space wavelength *λ*_0_ = 700 nm for an infinite Au nanorod metamaterial with *p* = 0.5 for (a) ordinary, TE, and (b) extraordinary, TM, modes. In the elliptic regime (a), the dispersion is bounded and corresponds to that of a typical anisotropic dielectric. The isofrequencies contours in the superstrate (air) and the substrate (glass) are also shown. (c) and (d) The mode position of a metamaterial slab (400 nm in thickness) shown as dots corresponding to intersection of the isofrequency contours of the infinite metamaterial with the quantized values of *k_z_* = *q*(π/*l*), where *q* = ±1, ±2, ±3,… resulting from the finite size of the slab in the *z*-direction. (e) and (f) Angular spectra of reflectance of the metamaterial slab as in (c) and (d) for *λ*_0_ = 700 nm calculated using TMM. The position of the modes obtained analytically is shifted to higher wavevectors due to the analytic model assumptions, influencing the confinement of the modes.

For a homogenized metamaterial described by EMT and neglecting nonlocal corrections, the propagation of extraordinary waves, in which the electric field is solely polarized along the long axis of the rods, is prohibited below the effective plasma frequency (hyperbolic regime), as expected for conductors. However, as a result of the anisotropy of the metamaterial, electron plasma oscillations may still give rise to bulk plasmon-polaritons propagating in the metamaterial below the plasma frequency in directions determined by the dispersion relation, 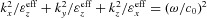, where *k_x,y_* and *k_z_* are the wavevector components along the *x*, *y* and *z* directions, respectively, *ω* is the electromagnetic field frequency, and *c*_0_ is the speed of light in vacuum. In a microscopic consideration of the plasmonic nanorod metamaterial studied here, these bulk plasmon-polaritons arise from interacting CSPs supported by individual nanorods forming the metamaterial 18, but can also be observed in multilayered metal–dielectric–metal metamaterials, where they arise from interacting smooth-film SPP modes 20–23. The isofrequency contours of the metamaterial dispersion for TE (elliptic) and TM (hyperbolic) modes show striking differences in the allowed wavevector ranges, which determines the dissimilar behavior of these modes in both the infinite metamaterial as well as in a metamaterial slab (Fig.3). In particular, for any given frequency, an elliptic TE mode can only be either unbound, leaky or waveguided (the example shown in Fig.3a), depending on the respective position of the metamaterial permittivity isofrequency contour with respect to that of the substrate and superstrate, while a hyperbolic TM mode may be of all three types at the same frequency (Fig.3b).

### 2.2 Mode structure of a metamaterial slab

The modes supported by a metamaterial slab, when one (*z*-) dimension is taken to be finite, were studied for a metamaterial slab placed on a silica substrate (*n* = 1.5) and with air as a superstrate. The slab geometry quantizes the *z*-component of the wavevector of the infinite metamaterial to values determined by the mode order and slab thickness, such as *k_z,_* = *q*(π/*l*). The modal behavior at a given frequency is then determined by the wavevectors satisfying both the quantization condition and bulk metamaterial dispersion (Figs.3c and d). These solutions have an *x*-component *k_x_*(*k_z_*,*q*) of the wavevector associated to each solution *k_z_* corresponding to the propagation constant *β* = *k_x_*(*k_z_*,*q*) of mode *q* in the slab. In this instance, the elliptic dispersion allows for a finite number of solutions with one unbound mode corresponding to a solution for the propagation constant within the isofrequency contour of both superstrate and substrate, as well as 3 waveguided modes. In contrast, the hyperbolic dispersion allows modes for any *q*-value, with practical limits eventually imposed by both losses and the geometry of the nanorod composite as the EMT breaks down for wavevectors near the boundary of the Brillouin zone. For the considered example (Fig.3d), the modes are present both within the light lines (Fabry–Perot modes) and confined to the metamaterial (waveguided modes).

The mode structure of the metamaterial slab can be numerically simulated using the transfer matrix method (TMM) approach in the complex incidence angle formulation 24. In this approach, both reflected and transmitted intensities from the 3-layer substrate/metamaterial/superstrate system are calculated using the effective medium description for the metamaterial. The angle of incidence of a plane wave in the substrate varies from 0 to π/2 (the angles corresponding to unbound and leaky modes in the substrate) and then to the complex incidence angles *θ* for which sin(*θ*) > 1 in order to cover the waveguided mode-dispersion region.

**Figure 4:**
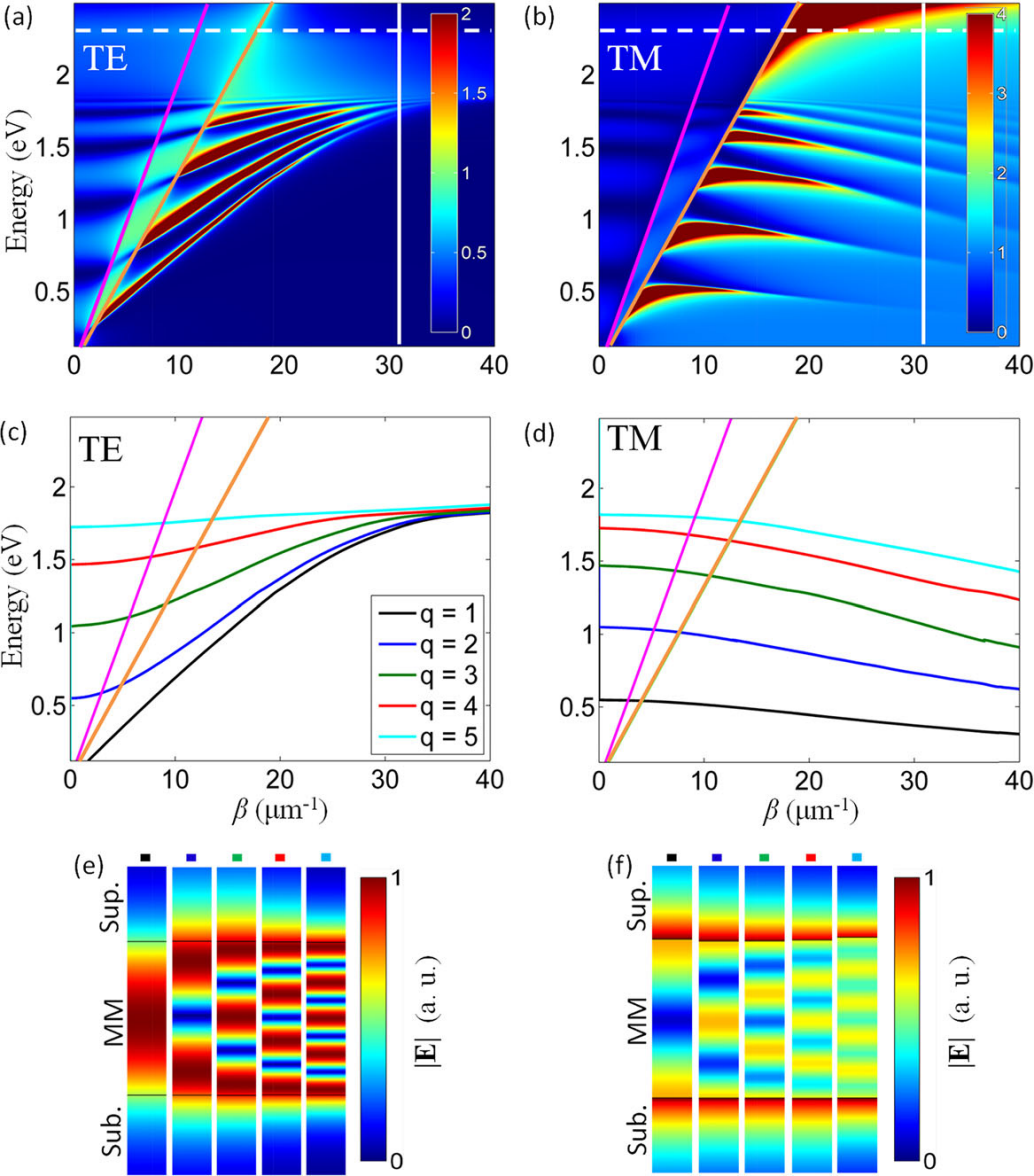
Reflection dispersion of the metamaterial slab calculated using the complex-angle TMM method (a) and (b) and analytic model (c) and (d) for a waveguide of 400 nm thickness with *p* = 0.5: (a) and (c) TE and (b) and (d) TM modes. The analytic model shows the 5 lowest (1 ≤ *q* ≤ 5) modes. The dashed line indicates the effective plasma frequency. The vertical line corresponds to the boundary of the Brillouin zone of the metamaterial realization when considering a square lattice of nanorods. The modes in (a) and (b) correspond to the minima of reflectance. Light lines in the superstrate (magenta) and the substrate (orange) are also shown. (e) and (f) The spatial distributions of the norm of the electric field for the *q* = 1–5 modes as obtained from the eigenmode simulations for (e) TE and (f) TM modes.

Using TMM, the mode dispersion of a typical planar waveguide with a thickness of 400 nm and made of metamaterial comprised of 100 nm period and 40 nm radius nanorods was calculated via angle resolved reflection spectra (Figs.4a and b). A rich family of modes is observed for both TE and TM polarizations with distinctively different behavior. Modes in the dispersion correspond to reflectance minima where the incident light is coupled to the modes. The reflectance corresponding to the isofrequency contours is in a good agreement with the solutions from the mode quantization in the finite size slab, giving the same number of modes with a small discrepancy in their positions due to the overestimated mode confinement (approximated boundary conditions) in the analytic simulations (Figs.3e and f).

The complex mode structure of the slab emerges as a consequence of the spatial confinement of plasmon-polaritons in the slab and is associated with both cavity resonances and waveguided modes, above and below the light line in air, respectively. In the cavity regime, the dispersion of TM modes reveals discrete modes with very low or negative group velocity. These unbound modes are not confined to the metamaterial slab, being accessible to plane waves in both the substrate and the superstrate. In this regime, the metamaterial slab simply acts as a Fabry–Perot (FP) cavity for bulk plasmon-polaritons resulting in effects similar to those observed for cavity-polaritons in semiconductors. Between the light lines in the substrate and the superstrate, the modes are coupled to radiation in the substrate (leaky modes), while being evanescent at the metamaterial/superstrate interface. Due to this coupling, the modes are ‘‘strongly bent’’ near the light lines and may have positive, negative, or vanishing values of the group velocity (Fig.4b). The modes below the substrate-line are truly guided modes decaying exponentially in both substrate and superstrate. A similar analysis holds for TE-polarized modes, but in this regime the metamaterial slab acts as a typical anisotropic dielectric waveguide due to the orientation of the electric field normal to the nanorod axes (ordinary direction) and does not support bulk plasmon-polaritons. The marked difference between TE and TM modes is in the opposite sign of the group velocity. Neglecting a strongly dispersive behavior in the vicinity of the light lines, the TM modes always show either negative or vanishing group velocity (Fig.4b), while the group velocity of TE modes is always positive (Fig.4a).

In order to understand the observed mode structure, we adapted an analytic description of the dispersion of ordinary and extraordinary waves in a conventional anisotropic waveguide 25 to a hyperbolic metamaterial. Within this framework, a planar hyperbolic waveguide is considered in the *x*–*y* plane with phase-insensitive reflections at the metamaterial's boundaries. Given the 2D geometry of the waveguide, modes can be separated by TM and TE polarizations. The dispersions of TE and TM guided modes can then be expressed as

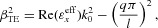
4

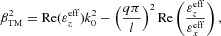
5where *k*_0_ *= ω/c*_0_, *q* is a positive integer referring to the mode number, and *l* is the thickness of the planar waveguide. The mode dispersions were calculated analytically for the first five modes *q* = 1–5 for TE and TM polarizations (Figs.4c and d). Although the analytical model does not take into account either substrate or superstrate, the analytical dispersions reproduce the TMM simulations with very good agreement in both the cavity and true-waveguide mode regimes, while not in the leaky mode regime. The analytical model formulation does not allow the fields to escape the waveguide and thus does not contain leaky modes.

For TM modes, the group velocity is negative below the light line in the substrate provided the condition 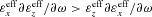 is satisfied. For bulk plasmon-polaritons in a hyperbolic metamaterial slab, this requirement is always fulfilled away from the resonances in 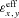 since 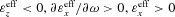 and 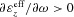. In the vicinity of the 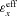
resonance, the term 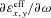 becomes negative and is to be taken into account to establish the hyperbolic behavior of the waveguide when the ENZ frequency is close to this resonance. However, in the epsilon near-zero (ENZ) regime, near the effective bulk-plasma frequency 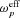, a different treatment is needed to take into account nonlocal effects 18,19, a scenario not considered here. It is important to emphasize that for typical metamaterial geometries, the condition for negative group velocity is satisfied for all TM-mode orders and any waveguide thickness.

The TE-mode dispersions are similar to those of dielectric waveguides and cavities with a group velocity 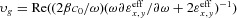. The sign of the group velocity is always positive except when the metamaterial exhibits anomalous dispersion 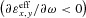; in this case, however, the losses near the 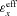 resonance (Fig.1c) prevent the existence of guided modes 8.

Both TE- and TM-mode dispersions are bounded by a high-frequency limit. TM modes converge, with increasing mode number *q*, to the effective plasma frequency of the metamaterials (dashed line in Figs.4a and b), above which bulk plasmon-polaritons do not exist. At the same time, TE modes are bounded by the EVL condition given by 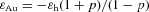 (Fig.2b). However, for each mode number, while TE modes demonstrate conventional behavior with a low-frequency cut-off determined by the mode number at small *β*_TE_, the behavior of TM modes is distinctly different and unusual. Each TM mode has a high-frequency cut-off for intermediate values of the propagation constant *β*_TM_ where the turning point of the dispersion curve is observed. No low-frequency cut-off is observed for increasing *β*_TM_ which is limited only by the Brillouin zone of the metamaterial realization. Thus, hyperbolic metamaterial slabs of deep-subwavelength thickness can act as multimode TM waveguides, a behavior that finds its origin in the inverse scaling law for hyperbolic metamaterial cavities 26.

**Figure 5:**
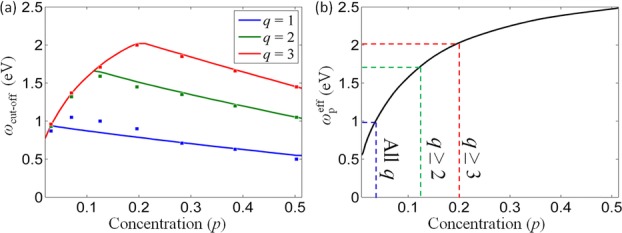
(a) The dependence of the high-frequency TM mode cut-off *ω*_cut-off_ on nanorod concentration, *p*, for *q* = 1–3 modes: (lines) analytic model, (squares) TMM simulations. (b) Effective plasma frequency, 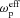, for the same nanorod concentration range. The frequency threshold below which the effective plasma frequency determines the mode cut-off is for 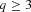 at *p* = 0.2, for 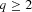 at *p* = 0.13, while for *p* < 0.04, the cut-off for all modes converges to the effective plasma frequency.

The high-frequency bound for TM-polarized modes can be obtained by solving Eq. 5: 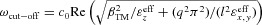, so that for frequencies higher than 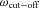, propagation along the hyperbolic slab waveguide for mode order *q* is prohibited as 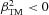. Figure5a shows the cut-off frequency dependence for the *q* = 1–3 TM modes for various nanorod concentrations obtained both analytically and with the TMM formalism. As the nanorod concentration falls, the mode cut-off frequency monotonously increases for *p* > 0.2 and then decreases for *p* < 0.2. The nonmonotonous behavior and, in particular, the decrease observed for smaller nanorod concentrations (smaller anisotropy of the metamaterial) is due to the decrease of the effective plasma frequency 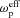 for decreasing nanorod concentrations. Since the existence of bulk plasmon-polaritons is determined by 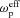, modes with 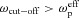 all adopt 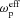 as the cut-off frequency. This occurs for *p* < 0.2 in Fig.5b. As a result, the mode density diverges as the frequency approaches the effective plasma frequency.

An eigenmode analysis of the mode structure of the 2D waveguide cavity allows the electromagnetic-field distributions associated with the modes to be mapped and confirms the analytical identification of the modes (Figs.4e and f). As the mode number increases, the field distributions show an increasing number of maxima/minima that are typical of standing-wave distributions inside the waveguide, as expected from the analytical calculations. Depending on the polarization, the waveguide acts either as a closed cavity with a field maximum close to its center (TE case) or as an open cavity with a field minimum at the center (TM case) of the slab waveguide. This is a direct consequence of boundary conditions due to the confinement along the *z*-direction 27.

## 3 Experimental results

The modes supported by a metamaterial slab were measured for the metamaterial placed on a silica substrate (*n* = 1.5) and with air as a superstrate. The waveguides were fabricated as described in detail in Ref. [28] and are formed by Au nanorods (∼100 nm period, ∼32 nm radius, giving an average concentration *p* ≈ 0.32) in an AAO matrix (inset in Fig.1a). The mode dispersion of the slab was determined experimentally via angle-resolved reflectance spectroscopy under plane-wave illumination through a semicylinder silica substrate, so that incident angles both smaller and larger than the angle of total internal reflection in silica were probed and the coupling to both cavity and leaky modes can be realized 29. Geometrical constraints in the experimental set-up determine the low-wavevector limit in the measured dispersions, while the high-wavevector limit is determined by the light line in the substrate.

**Figure 6:**
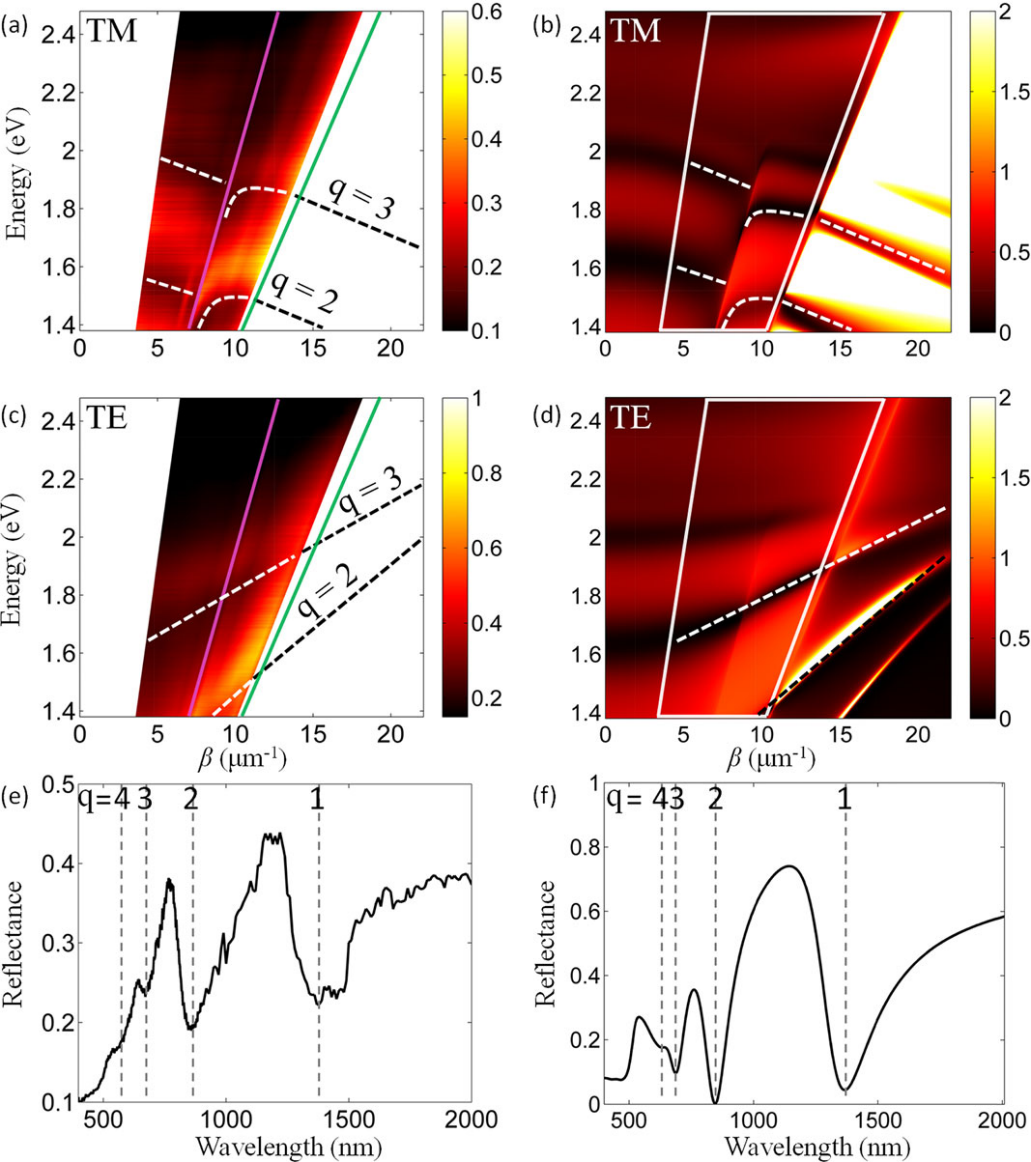
Experimentally measured (a) and (c) and simulated (b) and (d) reflectance dispersions for a 340-nm thick metamaterial slab with p ≈ 0.32 (the nanorod period is ∼100 nm and radius is ∼32 nm): (a) and (b) TM, (c) and (d) TE modes. The light lines in the substrate (silica, green) and the superstrate (air, magenta) are shown. The Au nanorods are embedded in an AAO matrix. The modes with *q* = 2 and 3 are tracked with dashed lines as a guide to the eye. The angular range measured in the experiment (20–75° in the substrate) is indicated with white boxes in (b) and (d). (e) and (f) Experimental and simulated reflectance spectra at the angle of 60^o^ for TM polarization as extracted from (a) and (b). The first four modes (*q* = 1–4) are indicated with dashed lines.

The measured and simulated reflectance dispersions reveal both cavity resonances and leaky modes, above and below the light line in air, respectively (Fig.6). More precisely, above the air-line, the dispersion of the TM modes reveals discrete modes with negative group velocity (Fig.6a). Between the light lines in the superstrate and substrate, the modes are coupled to the modes radiating in the substrate (leaky modes) while being evanescent at the metamaterial/air interface. Due to this coupling the modes (indicated with curved dashed lines in Figs.6a and b) can have positive, negative, or vanishing small group velocity. The truly waveguided modes, present below the substrate-line, are not accessible experimentally in the configuration used in the experiment but had been examined analytically and numerically in Section. A similar analysis for the dispersion of TE modes (Figs.6c and d) shows that the guided modes exhibit positive group velocity in all regimes. For these modes, as was discussed above, due to the orientation of the electric field along the *y*-axis only (normal to the nanorod axis), bulk plasmon-polaritons are not excited and the metamaterial slab acts as an anisotropic dielectric waveguide. It is thus possible to flip the sign of the group velocity of guided signals simply by altering the polarization of the coupled light.

Based on the analytical model (Eqs. 4 and 5), we can now identify the modes in the experimental reflectance as modes *q* = 1 to *q* = 3 (Fig.6). For the TM modes, as the mode order increases, both the group velocity and group-velocity dispersion decrease, with the *q* = 3 mode having negative group velocity even between the substrate and superstrate light lines. In general, the experimental dispersions for the modes with *q* = 2 and *q* = 3 (Figs.6a and c) are in satisfactory agreement with the TMM modelled dispersions (Figs.6b and d), further indicating that EMT is still applicable for the metamaterial parameters and propagation constants considered, as long as the frequency is away from the effective plasma frequency. It is important, however, to explain the discrepancies that occur for higher mode orders. It is evident from comparison of Figs.6e and f that the *q* = 4 mode is not observed experimentally. The reason for this is a combination of higher loss and mode density for increasing *q*'s at higher frequencies.

For practical purposes one can analyze the behavior of the modal effective index for TM waveguided modes (*β*>*ω*/*c*_0_) in a planar hyperbolic metamaterial, which can be expressed as 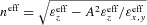, where 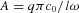. From this expression it is clear that the effective index increases with *q* and decreases with *l* for a given frequency, in contrast to TE modes’ effective index that exhibits the opposite behavior. As an example, for a telecom wavelength of 1.5 μm with *p* = 0.5 and *l* = 500 nm, the metamaterial slab supports multiple TM waveguided modes, with the real part of the effective index taking values of about 2, 9 and 13 for *q* = 2, 3, and 4, respectively (the TM mode with *q* = 1 is guided only at longer wavelength for these metamaterial parameters due to the unusual high-frequency cut-off discussed above). At the same frequency, for the same nanorod concentration *p* and *l* = 300, 400 and 500 nm, *n*^eff^ takes values of about 10, 6, and 2 for *q* = 2, respectively. This strong inter-relationship of metamaterial parameters and effective index provides a flexible way for modal design throughout the visible and infrared spectral ranges. Taking into account the wavevector cut-off due to the metamaterial realization (the Brillouin zone) and the high-frequency cut-off of the TM modes, the wavelength range of around 2400–3100 nm represents the single-mode guiding regime for *p* = 0.5, corresponding to a waveguide thickness range of only *λ*_0_/6–*λ*_0_/8. The energy confinement for the fundamental mode (*q* = 1) depends on the operating frequency, but, when measured as the ratio between the power flow inside the waveguide to the total power flow of the mode, is of the order of 40% at a wavelength of 2480 nm. This ratio increases for higher-order modes, as TM modes become increasingly confined to the waveguide. The dissipative nature of the hyperbolic waveguide results in increasing losses for higher-order modes, as their confinement in the guide increases.

## 4 Conclusions and outlook

We have investigated, experimentally and theoretically, the mode structure of finite-thickness hyperbolic metamaterial slabs. Similar to planar-cavity exciton-polaritons in semiconductors, planar hyperbolic metamaterial slabs support bulk plasmon-polaritons for TM polarization, which in different regimes result in planar-cavity modes or waveguided modes. TE-polarized modes are similar to anisotropic dielectric waveguide modes. It was shown that the spectral range of hyperbolic waveguided modes is bounded from the high-frequency side by the effective plasma frequency of the metamaterial. TM waveguided modes have negative group velocity and an unusual high frequency cut-off with no cut-off for high propagation constants, limited only by the Brillouin zone of the metamaterial realization. The negative group velocity as well as its dispersion can be controlled by varying the anisotropy of the metamaterial, i.e., the nanorod concentration. For the nanorod metamaterial studied, TM modes are slow modes with *υ*_g_ down to –0.03*c*_0_ and have a dispersion as low as 0.02 ps^2^/mm. Single-mode guiding can be achieved in planar waveguides of *λ*_0_/6–*λ*_0_/8 thickness, depending on the anisotropy of the metamaterial. These properties of hyperbolic metamaterial waveguides are interesting for designing integrated deep-subwavelength sensors, nonlinear and active nanophotonic devices, quantum information processing and optical data-storage components.

Planar hyperbolic metamaterial waveguides may be important for designing integrated deep-subwavelength chemical and biosensors, as well as nonlinear and active nanophotonic devices, and for quantum information processing. Careful control of both group- and phase-velocity dispersion is an important step towards the engineering of spontaneous emission properties and may lead to new nanoscale laser sources, including those based on slow-light properties. The fact that a slow-light regime can be achieved in a tunable and controllable environment opens up new possibilities in optical communications such as network buffering, data synchronization and pattern correlation.
